# Advancing microbiome research with machine learning: key findings from the ML4Microbiome COST action

**DOI:** 10.3389/fmicb.2023.1257002

**Published:** 2023-09-25

**Authors:** Domenica D’Elia, Jaak Truu, Leo Lahti, Magali Berland, Georgios Papoutsoglou, Michelangelo Ceci, Aldert Zomer, Marta B. Lopes, Eliana Ibrahimi, Aleksandra Gruca, Alina Nechyporenko, Marcus Frohme, Thomas Klammsteiner, Enrique Carrillo-de Santa Pau, Laura Judith Marcos-Zambrano, Karel Hron, Gianvito Pio, Andrea Simeon, Ramona Suharoschi, Isabel Moreno-Indias, Andriy Temko, Miroslava Nedyalkova, Elena-Simona Apostol, Ciprian-Octavian Truică, Rajesh Shigdel, Jasminka Hasić Telalović, Erik Bongcam-Rudloff, Piotr Przymus, Naida Babić Jordamović, Laurent Falquet, Sonia Tarazona, Alexia Sampri, Gaetano Isola, David Pérez-Serrano, Vladimir Trajkovik, Lubos Klucar, Tatjana Loncar-Turukalo, Aki S. Havulinna, Christian Jansen, Randi J. Bertelsen, Marcus Joakim Claesson

**Affiliations:** ^1^Department of Biomedical Sciences, National Research Council, Institute for Biomedical Technologies, Bari, Italy; ^2^Institute of Molecular and Cell Biology, University of Tartu, Tartu, Estonia; ^3^Department of Computing, University of Turku, Turku, Finland; ^4^Université Paris-Saclay, INRAE, MetaGenoPolis, Jouy-en-Josas, France; ^5^JADBio Gnosis DA S.A., Science and Technology Park of Crete, Heraklion, Greece; ^6^Department of Computer Science, University of Crete, Heraklion, Greece; ^7^Department of Computer Science, University of Bari Aldo Moro, Bari, Italy; ^8^Department of Biomolecular Health Sciences (Infectious Diseases and Immunology), Faculty of Veterinary Medicine, Utrecht University, Utrecht, Netherlands; ^9^Center for Mathematics and Applications (NOVA Math), NOVA School of Science and Technology, Caparica, Portugal; ^10^UNIDEMI, Department of Mechanical and Industrial Engineering, NOVA School of Science and Technology, Caparica, Portugal; ^11^Department of Biology, University of Tirana, Tirana, Albania; ^12^Department of Computer Networks and Systems, Silesian University of Technology, Gliwice, Poland; ^13^Systems Engineering Department, Kharkiv National University of Radio Electronics, Kharkiv, Ukraine; ^14^Department of Molecular Biotechnology and Functional Genomics, Technical University of Applied Sciences Wildau, Wildau, Germany; ^15^Department of Microbiology, Universität Innsbruck, Innsbruck, Austria; ^16^Department of Ecology, Universität Innsbruck, Innsbruck, Austria; ^17^Computational Biology Group, Precision Nutrition and Cancer Research Program, IMDEA Food Institute, CEI UAM+CSIC, Madrid, Spain; ^18^Department of Mathematical Analysis and Applications of Mathematics, Faculty of Science, Palacký University, Olomouc, Czechia; ^19^BioSense Institute, University of Novi Sad, Novi Sad, Serbia; ^20^Molecular Nutrition and Proteomics Research Laboratory, Department of Food Science, University of Agricultural Sciences and Veterinary Medicine of Cluj-Napoca, Cluj-Napoca, Romania; ^21^Department of Endocrinology and Nutrition, Virgen de la Victoria University Hospital, the Biomedical Research Institute of Malaga and Platform in Nanomedicine (IBIMA-BIONAND Platform), University of Malaga, Malaga, Spain; ^22^Department of Electrical and Electronic Engineering, University College Cork, Cork, Ireland; ^23^Chemistry and Pharmacy Department, University of Sofia, Sofia, Bulgaria; ^24^Computer Science and Engineering Department, Faculty of Automatic Control and Computers, University Politehnica of Bucharest, Bucharest, Romania; ^25^Department of Clinical Science, University of Bergen, Bergen, Norway; ^26^Department of Computer Science, University Sarajevo School of Science and Technology, Sarajevo, Bosnia and Herzegovina; ^27^Swedish University of Agricultural Sciences, Department of Animal Breeding and Genetics, Uppsala, Sweden; ^28^Nicolaus Copernicus University Torun, Torun, Poland; ^29^Computational Biology, International Centre for Genetic Engineering and Biotechnology, Trieste, Italy; ^30^Verlab Research Institute for BIomedical Engineering, Medical Devices and Artificial Intelligence, Sarajevo, Bosnia and Herzegovina; ^31^University of Fribourg and Swiss Institute of Bioinformatics, Fribourg, Switzerland; ^32^Department of Applied Statistics and Operations Research and Quality, Universitat Politècnica de València, València, Spain; ^33^British Heart Foundation Cardiovascular Epidemiology Unit, Department of Public Health and Primary Care, University of Cambridge, Cambridge, United Kingdom; ^34^Victor Phillip Dahdaleh Heart and Lung Research Institute, University of Cambridge, Cambridge, United Kingdom; ^35^Department of General Surgery and Surgical-Medical Specialties, School of Dentistry, University of Catania, Catania, Italy; ^36^Ss. Cyril and Methodius University, Skopje, North Macedonia; ^37^Institute of Molecular Biology, Slovak Academy of Sciences, Bratislava, Slovakia; ^38^Faculty of Technical Sciences, University of Novi Sad, Novi Sad, Serbia; ^39^Finnish Institute for Health and Welfare, Helsinki, Finland; ^40^Institute for Molecular Medicine Finland, FIMM-HiLIFE, Helsinki, Finland; ^41^Biome Diagnostics GmbH, Vienna, Austria; ^42^Institute of Science and Technology Austria (ISTA), Klosterneuburg, Austria; ^43^University of Bergen, Bergen, Norway; ^44^School of Microbiology & APC Microbiome Ireland, University College Cork, Cork, Ireland

**Keywords:** microbiome, machine learning, artificial intelligence, standards, best practices

## Abstract

The rapid development of machine learning (ML) techniques has opened up the data-dense field of microbiome research for novel therapeutic, diagnostic, and prognostic applications targeting a wide range of disorders, which could substantially improve healthcare practices in the era of precision medicine. However, several challenges must be addressed to exploit the benefits of ML in this field fully. In particular, there is a need to establish “gold standard” protocols for conducting ML analysis experiments and improve interactions between microbiome researchers and ML experts. The Machine Learning Techniques in Human Microbiome Studies (ML4Microbiome) COST Action CA18131 is a European network established in 2019 to promote collaboration between discovery-oriented microbiome researchers and data-driven ML experts to optimize and standardize ML approaches for microbiome analysis. This perspective paper presents the key achievements of ML4Microbiome, which include identifying predictive and discriminatory ‘omics’ features, improving repeatability and comparability, developing automation procedures, and defining priority areas for the novel development of ML methods targeting the microbiome. The insights gained from ML4Microbiome will help to maximize the potential of ML in microbiome research and pave the way for new and improved healthcare practices.

## 1. Introduction

In the recent decade, the human microbiome has been characterized in great detail in several large-scale studies as a critical player in many human diseases and conditions. As more associations between the microbiome and disease phenotypes are elucidated, the research focus is expected to shift towards identifying the microbiome-related biomarkers for disease diagnostics, prognostics, and therapeutics (Manor et al., 2020). Nevertheless, microbiome data analysis is challenging due to its intrinsic characteristics like compositional nature, high dimensionality (often more features than samples), technical variability, missing data, and integration needs. Another challenge in microbiome data analysis is the interpretation of statistical models, as microbiome data often contains many highly correlated variables. Machine Learning (ML) methods offer great potential to further progress microbiome science, but these obstacles first need to be mitigated. Thus, a dynamic collaboration between microbiome and ML researchers is pivotal. Some initiatives have made more general efforts to provide ML guidelines and standard recommendations for data management, preprocessing, analysis and integration, like the ELIXIR Machine Learning Focus Group^1^ (Walsh et al., 2021) or the ISO committees (ISO/TC 276 Biotechnology; ISO/IEC JTC 1/SC 42 Artificial intelligence; ISO/IEC TS 4213:2022 Assessment of Machine Learning Classification Performance).^2^

Moreover, while not explicitly focused on ML, the ongoing International Human Microbiome Coordination and Support Action (IHMCSA^3^) maps the necessary steps for innovation and builds consensus on priorities and means for the future of microbiome science and its translation. This includes standardization of microbiome analysis methods, which in its extension, also includes ML. The adoption of FAIR principles (Findable, Accessible, Interoperable, Reproducible) by ML tools and models is also being approached by FAIR4ML.^4^ However, these ML-focused initiatives are general and do not consider microbiome data or their characteristics. Scientific fields for which the study of human microbiota is essential, such as health and nutrition, have highlighted the need to join forces in the standardization and interoperability to integrate microbiome data with ML tools (Walsh et al., 2021; Balech et al., 2022). The European Cooperation in Science and Technology (COST) Action ML4Microbiome^5^ - Statistical and machine learning techniques in human microbiome studies (CA18131) - started in 2019 to create a productive symbiosis between discovery-oriented microbiome researchers and data-driven ML experts to prompt the optimization and standardization of the best practice use of ML techniques for human microbiome research. Up to now, ML4Microbiome has gathered researchers from 35 different European countries, attracted and trained a large number of young scientists and published various scientific articles. The following sections discuss the Action’s network research topics, elaborate on their relevance to the research challenges, and briefly overview more relevant achievements.

### 1.1. The ML4Microbiome action plan and challenges

**Figure 1:**
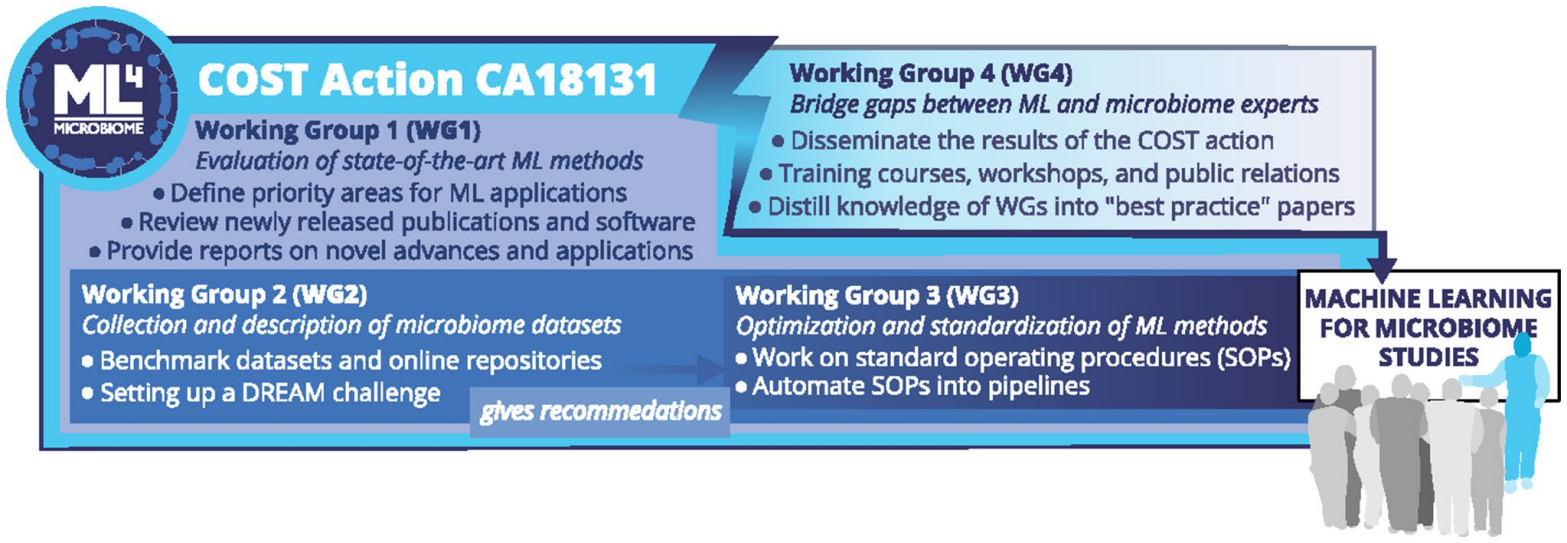
ML4Microbiome COST Action’s Working Groups. The figure shows the organization of the COST Action ML4Microbiome in four Working Groups (WGs), each committed to specific objectives. WG1 evaluated the state-of-the-art ML methods and software applied in human microbiome studies to define priority areas for novel machine learning and statistics applications that better address the specific challenges of human microbiome analysis. WG2 aimed to collect (from external projects and repositories) datasets describing microbiomes and characteristics of the underlying cohorts to test which ML methods are most robust and comparable, to provide more optimized parameters for the use of these methods, to develop novel ML methodologies and to implement a DREAM Challange on clinical data. WG3 investigated opportunities for automating the established Standard Operating Procedures (SOPs) into pipelines for translational use by clinicians and non-experts. WG4 goal was to bridge existing gaps between ML (bioinformaticians, statisticians, computer-science scientists) and microbiome experts through the organization of meetings, workshops, conferences, training schools, dissemination and communication activities.

To accomplish its goals, the ML4Microbiome network has designed an operational plan based on the coordinated and integrated work of four working groups (WGs), each addressing specific objectives (Figure 1). Several specific technical challenges have been identified (Moreno-Indias et al., 2021). Sequence-based microbiome studies use different types of data (16S rRNA gene or ITS amplicons/shotgun metagenomics or metatranscriptomics). Due to their different origin and types, separate modeling approaches are required. Moreover, microbiome data have large inter-individual variability and elevated noise levels, which Gaussian or log-normal models do not approximate well, providing challenges for traditional statistical methodologies (Voigt et al., 2015). There are more features than samples/observations (e.g., 100 studied humans may each have 1,000 microbial species and 1,000,000 microbial genes). This makes the application of ML methods challenging due to the curse of dimensionality, whereby huge data sparseness compromises the identification of data patterns or rules. Microbiome features often exhibit a complex dependency structure (taxonomic hierarchy or genes co-varying in abundance as encoded on the same genome, plasmid or phage). The relative abundance of each taxon is inherently related to the abundance of all other taxa, making it difficult to identify differentially abundant taxa (Weiss et al., 2017).

Microbial communities are also highly diverse, with many low-abundance taxa present only in a few samples. This can lead to high sparsity levels in the data, making it difficult to estimate the abundance of rare taxa accurately. Microbiome data is often compositional because most current studies have access only to the relative abundance of one microbial taxon (Gloor et al., 2017). In such cases, the abundance of one taxonomic group is constrained by the abundance of other taxonomic groups in the sample. Analyzing microbiome data as compositional data requires specific statistical approaches that account for this characteristic and address its unique challenges. Class sizes may be imbalanced (e.g., fewer disease samples than controls) (Ahlawat et al., 2021). An imbalanced class distribution coupled with high dimensional data poses a significant drawback for applying ML algorithms and results (Kim and Kim, 2018).

### 1.2. The current state of ML applications for microbiome data analysis

To assess the state-of-the-art of ML applications in microbiome data analysis, Working Group 1 (WG1) conducted a literature review accessible across the web application Machine Learning meTagenomic REsearch Scraper (MoLTRES^6^). The main aim of the tool is to provide a user-friendly interface for centralized searching and storing ML studies on human microbiome data, encompassing feature selection, biomarker identification, disease prediction and treatment. The review highlighted a steady increase in the utilization of ML methods for human microbiome analysis in recent years. Most studies (>70%) using ML employed 16S rRNA gene amplicon sequencing data as the input data type, while 27% used only shotgun metagenome data. The most frequently used ML methods were random forest, logistic regression, and support vector machines. While the former method remained the most popular, the use of logistic regression and support vector machine algorithms has increased. These results were published by ML4Microbiome (Marcos-Zambrano et al., 2021), and subsequent updates by WG1 members were incorporated into MoLTRES.

### 1.3. Benchmark datasets and online repositories

When analyzing microbiome data, it is often helpful to create reference datasets to test existing or new ML tools, whether separate or combined. The importance of validation sets and gold standards is largely discussed in Papoutsoglou et al. (2023). Pasolli et al. (2016) have demonstrated that the performance of ML models may vary substantially depending on the disease addressed in the dataset. For this reason, Working Group 2 (WG2) and Working Group 3 (WG3) decided to establish a benchmark dataset based on a single disease for which a reasonable amount of public data was available. The choice has been made on colorectal cancer, for which 2090 human stool samples have been characterized by shotgun metagenomic sequencing from 13 public cohorts spanning nine countries. This data provides the gut microbiota composition in healthy controls and patients with adenoma or colorectal cancer. The shotgun dataset is publicly available (Barbet et al., 2022). To complement the shotgun-based benchmark dataset, a 16S rRNA gene sequencing dataset of samples from colorectal cancer patients and available metadata was curated by WG3 members, including *n* = 709 samples from previous studies (Zackular et al., 2014; Zeller et al., 2014; Baxter et al., 2016). The final curated dataset is available in the Zenodo repository (Marcos-Zambrano Judith, 2022). WG2 was also responsible for defining and evaluating the ML4Microbiome DREAM Challenge.^7^ The challenge was designed to predict incident heart failure risk in a large population-based study of Finnish adults, FINRISK 2002 (Salosensaari et al., 2021), using a combination of gut microbiome data and clinical variables. The results of this DREAM Challenge, completed by 32 participants (seven teams), will be published separately (manuscript in preparation).

### 1.4. Optimization and standardization of machine learning methods - challenges and solutions

For the optimization and standardization of ML methods, WG3 considered a typical ML workflow that starts after microbiome-related profiles are organized in a two-dimensional table format of features, such as MSP (Metagenomic Species) or Amplicon Sequence Variants (ASV) tables for shotgun or 16S rRNA amplicon data, respectively. This process involves the following steps, (a) data preprocessing (e.g., normalization, filtering), (b) feature selection, (c) predictive modeling, and (d) performance estimation. Our objective was to address the challenges associated with each of these steps considering diverse algorithms, their combinations, and our capacity to interpret and explain their results. Although computational simulations may help estimate expectations and variability under uncertain situations (see, e.g., Gao et al., 2023), we explored benchmark data from the public domain spanning 16 different cohorts from nine countries and derived several noteworthy conclusions.

In data preprocessing, a major challenge lies in selecting the appropriate approaches due to variations in sampling depth, data sparsity (represented by an excess of zeros in the tables) and data compositionality. To first mitigate sampling variability, rarefaction is sometimes used to remove samples. However, this has remained a controversial practice since rarefaction reduces statistical power (McMurdie and Holmes, 2014), but it also provides the means to deal with uncertainties related to variations in read counts that are otherwise challenging to control (Schloss, 2023). Alternatively, researchers incorporate the differences in library size (number of reads per sample) as covariates in the models designed to consider offsets. Sparsity further hampers models that rely on Gaussian assumptions (e.g., linear models), while other models do not have distributional assumptions (e.g., Random Forests, Boosting models). In addition, this sparsity can lead to near-zero variance predictors that turn out to be zero variance predictors during the cross-validation process. Our results indicated that filtering out rare features and removing near-zero variance ones is a successful strategy, outperforming imputation methods in logarithmic transformations. Moreover, standard sequencing techniques cannot capture the total number of bacterial species but only their proportions. For this reason, compositional analysis is the appropriate mathematical framework (Gloor et al., 2017), but its application and impact on ML models are still actively investigated (Greenacre and Blasco, 2021). For example, we found that the CLR transformation can be useful; however, its generalizability to other data sets should be investigated. Therefore, due to the huge variability of approaches and frequently evolving methodologies, we are against giving precise and definitive recommendations.

For feature selection and predictive modeling, the primary challenges revolve around the high dimensionality of the data and the complex interactions inherent to microbial species, including co-occurrence and partial correlation. Building models that incorporate the thousands of microbiome features in a multivariate manner (e.g., principal component regression, partial least squares models) while maintaining predictive performance is undeniably challenging. Boosting or Random Forest models often provided the best performances. Interestingly, using the JADBio autoML approach, we observed that multivariate feature selection through the Statistically Equivalent Signatures algorithm combined with Random Forests could yield an optimal balance between performance and results interpretability and explainability (Tsamardinos et al., 2022). We also emphasize that appropriate performance estimation protocols are crucial to avoid overestimated conclusions and misleading insights. A summary of methods that can be used for each one of the steps of the ML workflow is reported in Table 2 of Papoutsoglou et al. (2023).

A novel multi-view learning method was developed based on boosting and multi-armed bandits. The goal was to simultaneously exploit (possibly incomplete) 16S and shotgun data about the same individuals, as well as the features identified through multiple preprocessing pipelines. The obtained results showed significant benefits towards an automated selection and exploitation of multiple views/pipelines for the analysis of microbiome data (manuscript submitted).

### 1.5. Community building, networking and training: the three key to success

The specific commitments of Working Group 4 (WG4) were to bring networking and training opportunities for emerging talents and thereby strengthen and build up an excellent scientific and technological community, including both ML and microbiome researchers. Providing people with opportunities (internal meetings, conferences and workshops) to discuss and present ideas and experiences was pivotal for establishing collaborations, developing new multidisciplinary interactions, attracting young researchers and providing them with opportunities for their scientific and professional career growth. Thanks to these activities, and despite the interference of the COVID-19 pandemic, the ML4Microbiome network expanded from the initial 24 member countries to 35 (55% from COST Inclusiveness Target Countries), and participants from 57 to 169, among which 48% represented by Young Researchers and Innovators (<40 years). Some could benefit from Short Term Scientific Mission (STMS) grants (16 in total) to work with research teams in different countries on ML4Microbiome-related projects with the view to publish the results of their activities in peer-reviewed journals.^8^

In terms of publication output, to date ML4Microbiome members have published work on specific ML applications for particular diseases, such as Cancer Diagnostics and Therapeutics (Cekikj et al., 2022), classification of patients with Celiac Disease (Arcila-Galvis et al., 2022), Coronary Artery Disease Risk Prediction (Vilne et al., 2022), novel paradigms in human gut microbiome metabolism (Bidkhori et al., 2021), Parkinson’s disease (Rosario et al., 2021), Type 2 Diabetes (Ruuskanen et al., 2022), oral and related gut diseases (Di Stefano et al., 2023), along with systematic or scoping reviews on ML applications on microbiome data (Tonkovic et al., 2020; Marcos-Zambrano et al., 2021) and its challenges and solutions (Moreno-Indias et al., 2021) of which all are available from the complete list of the Action’s publications on the ML4Microbiome website.

Training schools (TSs) were organized to provide young researchers with the proper background knowledge and hands-on training in MLs techniques applied to microbiome data. Four Training Schools were organized in four different countries, in which 19 trainers and 125 attendants participated over three-five days. Plenary blended learning sessions with keynote speakers were offered, along with high-level lectures covering specific ML-microbiome topics complemented by practical sessions and workshops. The different scientific and geographical backgrounds enhanced multidisciplinary discussions and promoted knowledge exchange between academics and industry participants, leading to scientific publications (Deutsch et al., 2021; Deutsch and Stres, 2021; Deutsch et al., 2022). This also helped trainers learn more about the real needs of young researchers in such a complex multidisciplinary research field, further sharpening the training methods for subsequent TSs. As a result, a syllabus was created, funded by one of Action’s STMS, to incorporate ML for microbiome analysis into microbiome MSc courses at various institutes,^9^ which previously only addressed read processing, clustering methods, diversity analysis and statistical analysis (manuscript in preparation). All the training material produced by ML4Microbiome, STMS reports, and presentations are freely available from the Action’s website (see Footnote 5).

## 2. Discussion

Currently, microbiome research faces a new bottleneck: its translation into a clinical context, addressing risk, diagnosis/prognosis, and monitoring the effectiveness of therapy. The benefits of such applications involve better methodologies for current bioinformatics challenges, such as species identification from microbiome sequencing data, robust methods for microbiome-derived predictive models or statistical causal inference, and integration of microbiome data with other omics (Feldner-Busztin et al., 2023), among many others (and the possible impact of such applications in the clinic). Statistical modelling and analysis of microbiome-related omics data involve applying various techniques and ML algorithms, which ultimately aim to identify associations (and ideally causality) between microbial taxa, functional genes, metabolites, and host factors (e.g., omics and biochemical variables) with health and disease outcomes. We have outlined the challenges of such analysis and highlighted the importance of developing and optimizing statistical methods and pipelines to handle microbiome data’s unique properties for accurate and reproducible microbiome research.

Somewhat disappointingly, albeit not unexpected, there is no unique ML approach to extract the hidden meaningful information beyond the massive microbiome data. Instead, combinations of ML tools seem to be the most promising approach coupled with knowledge of the parameters that need tuning. As we advance, the application of deep learning (DL), a particular component of ML, to microbiome analysis holds significant promise in understanding the intricate relationships between microbial communities and their functions, as well as their links to various diseases and phenotypes (Hernández Medina et al., 2022). We have, however, identified several challenges with implementing DL methods for microbiome data analysis, which can be extended to any ML model, that first need to be addressed. Firstly, the availability (abundance) and quality of microbiome samples and metadata currently limit the collection of large and diverse datasets for the training and validation of DL models, which are even more dependent on large sample sizes. Additionally, there is the issue of interpretability and explainability of DL models, which can restrict the biological insights and hypotheses that can be derived from them. Since many microbiome analysis applications are related to healthcare, the interpretation of the ML models becomes a priority issue, especially for non-ML experts. Without understanding how the decision was made and the specific reasons for the outcome, many physicians would hesitate to trust the ML results, which could have ethical or legal consequences. In response, Explainable AI (XAI) methods such as SHAP (Shapley Additive exPlanations), DeepSHAP, DeepLIFT, CXplain, and LIME (Lipton, 2016; Chen et al., 2022; Molnar, 2022) have been widely used in recent years. Analysis of microbiome data, such as personalized biomarker identification (Rynazal et al., 2023) and accurate predictions of phenotypes (Carrieri et al., 2021), have also been used to improve the understanding of disease mechanisms and microbiome associations. Nevertheless, XAI has some limitations as many of its models are highly complex and possess many parameters, making it difficult to define the factors that affect the explanation. A tradeoff between explainability and accuracy, which depends on the application area, within which it is determined how critical the accuracy of the model is for the end user.

As ML advances, it is also crucial to consider its ethical implications, particularly its use in clinical practice. One significant ethical consideration in ML and microbiome research is the potential for biased or discriminatory algorithms. It is imperative to ensure that the data sets used to train ML models are diverse and representative of the studied population (Mehrabi et al., 2021). Additionally, the sensitive nature of microbiome data, including health and genetic information and their associated metadata, raises privacy concerns and the need for informed consent (Shabani and Borry, 2018). Therefore, ethical guidelines for data collection, storage, and usage must be implemented to protect individual rights and maintain the integrity and validity of the research (Knoppers and Chadwick, 2005). As such, ML-enabled microbiome research must be conducted responsibly and ethically to ensure that the benefits are equitable, sustainable, and safe (Anomaly, 2017). The outcomes generated by numerous studies have already impacted the microbiome research community. Nevertheless, further advancing the field requires increasing collaborative efforts between microbiologists and ML experts, including stakeholders in non-governmental organizations, health sectors and industry once more standardized ML-microbiome applications start to become available. The main objective of the COST Action ML4Microbiome has significantly improved these opportunities. Thanks to this initiative, we have sown the seeds for a dynamic, interconnected, cross-disciplinary community that has already contributed to advancing research in the field, but with more to come.

## Data availability statement

The original contributions presented in the study are included in the article/supplementary material, further inquiries can be directed to the corresponding author.

## Author contributions

DD’E: Conceptualization, Supervision, Writing – original draft, Writing – review & editing, Visualization. JaaT: Writing – review & editing. LL: Writing – review & editing. MB: Writing – original draft, Writing – review & editing. GeP: Writing – review & editing. MiC: Writing – review & editing. AZ: Writing – review & editing. ML: Writing – original draft, Writing – review & editing. EI: Writing – original draft, Writing – review & editing. AG: Writing – review & editing. AN: Writing – original draft, Writing – review & editing. MF: Writing – review & editing. TK: Visualization, Writing – review & editing. EP: Writing – review & editing. L-MZ: Writing – original draft, Writing – review & editing. KH: Writing – review & editing. GiP: Writing – review & editing. AnS: Writing – review & editing. RamS: Writing – review & editing. IM-I: Writing – review & editing. AT: Writing – review & editing. MN: Writing – review & editing. E-SA: Writing – review & editing. C-OT: Writing – review & editing. RajS: Writing – review & editing. JasT: Writing – review & editing. EB-R: Writing – review & editing. PP: Writing – review & editing. NJ: Writing – review & editing. LF: Writing – review & editing. ST: Writing – review & editing. AlS: Writing – review & editing. GI: Writing – review & editing. DP-S: Writing – review & editing. VT: Writing – review & editing. LK: Writing – review & editing. TL-T: Writing – review & editing. AH: Writing – review & editing. CJ: Writing – review & editing. RB: Writing – review & editing. MaC: Funding acquisition, Project administration, Supervision, Writing – review & editing.

## Footnotes

^1^https://elixir-europe.org/focus-groups/machine-learning

^2^https://standards.globalspec.com/std/14568212/ISO/IEC%20TS%204213#:~:text=ISO%2FIEC%20TS%204213%20October%201%2C%202022%20Information%20technology,performance%20of%20machine%20learning%20models%2C%20systems%20and%20algorithms

^3^https://humanmicrobiomeaction.eu/

^4^https://www.rd-alliance.org/groups/fair-machine-learning-fair4ml-ig

^5^https://www.ml4microbiome.eu/

^6^http://imdeafoodcompubio.com/index.php/moltres/

^7^https://www.synapse.org/#!Synapse:syn27130803/wiki/616705

^8^https://www.ml4microbiome.eu/research-updates/publications-outputs/

^9^https://microbiome.github.io/OMA/
